# Impact of Chromatin Structures on DNA Processing for Genomic Analyses

**DOI:** 10.1371/journal.pone.0006700

**Published:** 2009-08-20

**Authors:** Leonid Teytelman, Bilge Özaydın, Oliver Zill, Philippe Lefrançois, Michael Snyder, Jasper Rine, Michael B. Eisen

**Affiliations:** 1 Department of Molecular and Cell Biology, California Institute of Quantitative Biosciences, University of California, Berkeley, California, United States of America; 2 Department of Molecular, Cellular and Developmental Biology, Yale University, New Haven, Connecticut, United States of America; 3 Howard Hughes Medical Institute, University of California, Berkeley, California, United States of America; Texas A&M University, United States of America

## Abstract

Chromatin has an impact on recombination, repair, replication, and evolution of DNA. Here we report that chromatin structure also affects laboratory DNA manipulation in ways that distort the results of chromatin immunoprecipitation (ChIP) experiments. We initially discovered this effect at the *Saccharomyces cerevisiae HMR* locus, where we found that silenced chromatin was refractory to shearing, relative to euchromatin. Using input samples from ChIP-Seq studies, we detected a similar bias throughout the heterochromatic portions of the yeast genome. We also observed significant chromatin-related effects at telomeres, protein binding sites, and genes, reflected in the variation of input-Seq coverage. Experimental tests of candidate regions showed that chromatin influenced shearing at some loci, and that chromatin could also lead to enriched or depleted DNA levels in prepared samples, independently of shearing effects. Our results suggested that assays relying on immunoprecipitation of chromatin will be biased by intrinsic differences between regions packaged into different chromatin structures - biases which have been largely ignored to date. These results established the pervasiveness of this bias genome-wide, and suggested that this bias can be used to detect differences in chromatin structures across the genome.

## Introduction

Chromatin packaging affects transcription, replication, and recombination in eukaryotic organisms [1]–[4]. Recent publications have also highlighted the impact of chromatin structure on rates and patterns of nucleotide substitution. Genes situated in heterochromatin of *Drosophila melanogaster* mutate faster than their euchromatic counterparts [5], silenced DNA of yeasts has increased rates of base-pair substitutions [6], and nucleosome-bound and linker DNA evolve at different rates in the Japanese killifish *Oryzias latipes* [7]. Given the influence of chromatin on so many biochemical processes *in vivo*, we wondered how the chromatin state of a locus might affect its behavior in experimental procedures. In particular, does chromatin introduce biases in the physical manipulations involved in the chromatin immunoprecipitation technique, and if so, are such effects exclusively confounding, or potentially useful?

The analysis was motivated by our prior results regarding DNA shearing at the silenced mating locus *HMR* of *Saccharomyces cerevisiae*. Silenced mating cassettes at the *HML* and *HMR* loci are the yeast version of heterochromatin. Regulatory sites, called silencers, flank *HML* and *HMR* in *S. cerevisiae*, and recruit the Sir proteins, which then spread throughout the loci and repress transcription (reviewed in [8]). We assayed DNA shearing in the presence and absence of Sir2, which is essential for silencing. At the global level, the extent of shearing across the genome was similar in Sir+ and Sir− strains, but when evaluated at the *HMR* locus specifically, shearing by sonication was quantitatively more extensive in Sir− cells relative to Sir+ cells (Özaydın B., submitted). Thus, a complex biological state of chromatin *in vivo* exercised an impact on physical manipulations of chromatin *in vitro*. This result led us to ask whether chromatin structures influence experimental results only at silenced mating cassettes, or more broadly in other heterochromatic regions, or even in euchromatin across the genome.

For the genome-wide analysis, we relied on data from the ChIP-Seq experiments that use high-throughput sequencing to map the binding of specific proteins or chromatin modifications across the genome [9], [10]. In particular, we examined the distribution of sequencing reads from input samples in which sheared chromatin was sequenced without being immunoprecipitated. Our computational analyses demonstrated that this control dataset contained an unexpected treasure-trove of information reflecting differences in the physical properties of DNA associated with different types of chromatin structures.

## Results

In chromatin immunoprecipitation and many other experimental genomic applications, DNA is physically sheared to produce small fragments prior to subsequent manipulations. If the shearing is not uniform, such that some regions of the genome are over-represented among long fragments, we reasoned that these regions would produce relatively fewer sequence reads. This would be due to a lower density of fragment ends, to size-selection prior to sequencing, and to biases for small fragments during the sequencing process. If broad domains of shearing-resistant chromatin exist in the yeast genome, we expected that such regions would be under-represented among the sequence reads in the input controls of ChIP-Seq experiments in which the sheared formaldehyde-crosslinked chromatin has not yet been fractionated by an antibody against a protein or modification of interest.

To explore this possibility, we mapped twelve million input-Seq reads to the *S. cerevisiae* genome. Throughout the manuscript, “input” refers to the sequence reads from this crosslinked and sheared non-immunoprecipitated DNA. To control for biases in sequencing and mapping, we also mapped nine million published reads from purified genomic DNA (“genomic”) that had also been sheared in preparation for deep sequencing [11]. In 100 base-pair sliding windows across the genome, we divided the median number of mapped input reads by the median number of mapped genomic reads for each window (Dataset S1). The median per-base coverage of the input DNA sequence reads was 16-fold, and for the genomic DNA sequence reads was 8-fold, giving a genome-wide ratio of 2. We then ranked all windows from least- to most-covered by input sequence reads, normalized by the genomic read counts.

### Bias against sequence reads in *HMR*, *HML*, and subtelomeric regions

**Figure 1:**
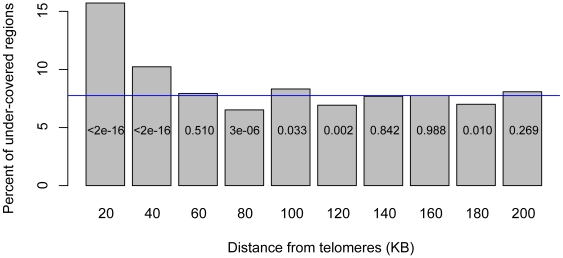
Distribution of input-Seq under-covered regions across chromosomes. Percent of regions with low input sequence coverage, as a function of distance from telomeres, in 20 KB intervals. The χ^2^ p-values for each 20 KB interval, comparing the fraction of under-covered regions in that interval to the under-covered fraction genome-wide are shown within each plot. The blue line indicates the average percent of under-covered regions, genome-wide (7.6%).

The 300 least-covered fragments (Table S1) included *HML* and *HMR*, the silenced mating cassettes, confirming our ability to detect areas that do not shear well due to silencing. Of the 300 regions, 159 (53%) were in subtelomeric regions (within 50 kilobase pairs of telomeres), where silencing proteins also form repressive chromatin [12]–[14]. As the total fraction of the *S. cerevisiae* genome that is subtelomeric is 13.4%, the proportion of under-covered DNA in subtelomeric regions was significantly enriched (p<10^−16^ by χ^2^-statistic). Across the genome, only the subtelomeric regions were unusually enriched in under-covered fragments (Figure 1).

### Over-representation of reads in telomeric repeats

**Figure 2:**
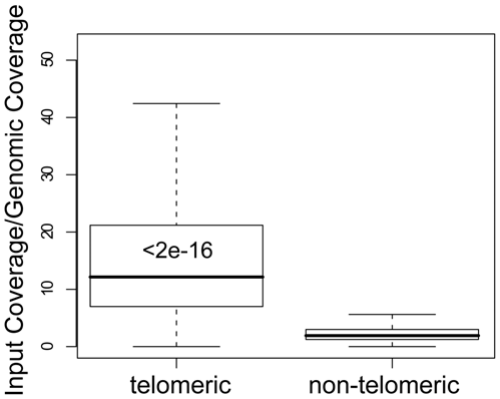
High input-Seq coverage in telomeres. Boxplots of input-Seq read coverage, normalized to non-crosslinked genomic reads, for telomeric and non-telomeric regions. Wilcoxon-Mann-Whitney p-value, comparing input coverage distribution of telomeric to genome-wide DNA, is shown within the telomeric boxplot. The top of each box in the boxplots indicates the 75% percentile, the bottom the 25% percentile, and the thick bar inside the box is the median. The whiskers extend out to the most extreme data point that is at most 1.5 times the interquartile range from the box.

With silent chromatin associated with under-sampling of input reads, we asked whether other chromatin states could distort the coverage in the opposite direction, resulting in an increased read density. Of the highest-covered 300 regions (Table S2), 138 (46%) were inside telomeres, even though telomeric DNA constitutes just 1.17% of the genome, as annotated in the *Saccharomyces* Genome Database [15]. The enrichment of coverage in telomeres was striking. The median input coverage inside telomeres was 128×, compared to 16× genome-wide. Almost no telomeric increase was observed for the genomic reads, where median telomeric coverage was 11×, compared to 8× genome-wide. The normalized coverage of telomeric DNA was almost completely non-overlapping with the rest of the genome (Wilcoxon-Mann-Whitney p<10^−16^) (Figure 2).

### High coverage over transcription factor binding sites and DNase I footprints

**Figure 3:**
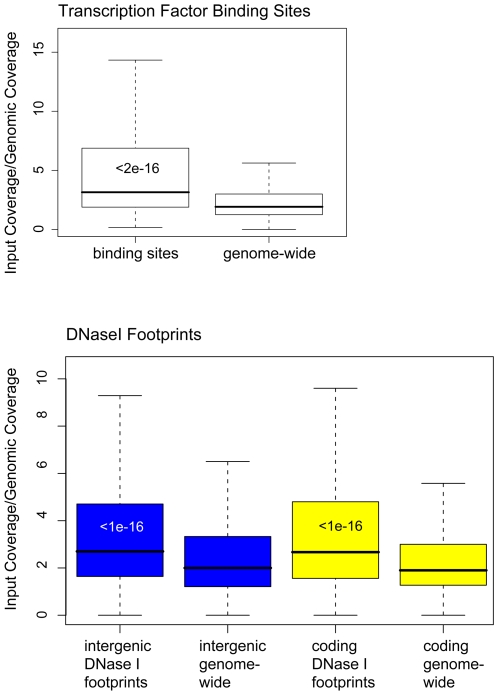
High input-Seq coverage across sites of protein-DNA interaction. Boxplots of input-Seq read coverage, normalized to genomic reads. The upper panel shows the boxplots for transcription factor binding sites and genome-wide input coverage. The lower panel shows the boxplots for DNase I-resistant footprints and genome-wide distributions of input sequence coverage. In blue are the boxplots of intergenic regions and in yellow the boxplots of coding regions. Wilcoxon-Mann-Whitney p-values, comparing input coverage distributions of binding sites or DNase I-footprinted site to genome-wide DNA, are shown within the boxplots.

Analyzing the hyper-covered locations, we noticed that many of the most enriched, non-telomeric loci were inside intergenic regions. Because regions upstream of genes tend to have high histone turnover and may be nucleosome-depleted [16]–[18], we hypothesized that the increased coverage of intergenic DNA may be due to the presence of DNA-binding proteins and their influence on nucleosome positioning and turnover. We analyzed read densities for 4,900 inferred transcription factor binding sites, conserved in closely-related yeast species and for which there is supporting evidence from ChIP-chip experiments [19]. The majority of these binding sites are likely to represent bona fide regulatory sites. The coverage over binding sites was much higher than over the rest of the intergenic regions (Figure 3, upper panel). Separating the binding sites by the corresponding transcription factor, input reads were high for almost all of the factors. Of the 37 transcription factors with 40 or more binding sites in the dataset, only two, Ste12 and Dig1, were not enriched in input-Seq coverage (Table 1).

**Table 1:** Input-Seq coverage of transcription factor binding sites.

Transcription Factor	Total Binding Sites in the Genome	median input-Seq reads
STE12	179	16
DIG1	161	17
FKH2	62	19
PHO2	90	19
TEC1	55	19
CIN5	82	20
DAL82	40	20
FKH1	61	20
MCM1	43	20
YAP6	65	20
MBP1	124	21
NDD1	61	21
ABF1	151	22
REB1	154	22
UME6	72	22
YAP7	50	22
GCN4	106	23
GLN3	52	23
RPN4	47	24
SWI5	91	25
GCR2	45	26
ACE2	40	29
FHL1	70	29
MSN2	81	29
MSN4	68	29
SWI6	164	29
CBF1	115	30
SWI4	143	31
NRG1	67	33
RAP1	66	35
SOK2	91	38
PHD1	172	40
HAP1	60	50
RCS1	54	52
AFT2	66	69
SKN7	130	82
SUT1	140	88

Transcription factors with 40 or more binding sites throughout the genome, per factor. The third column shows the median input-Seq read counts for all of the 100 bp windows that encompass the corresponding transcription factor's binding sites.

As an alternative test, we asked if input coverage was usually higher in sites of protein-DNA interaction. As a proxy for such regions, we used the putative interaction regions, based on the genome-wide *in vivo* DNase I footprinting study [20]. Indeed, coverage was significantly higher over the footprints, inside both coding and intergenic regions (Figure 3, lower panel).

### Transcription-dependent variation in sequence coverage of genes and promoters

In yeast and *Drosophila*, the rate of histone turnover in promoters is correlated with transcription levels of the adjacent genes [17], [18]. This rapid turnover, combined with our observations of high sequence coverage over binding sites and DNaseI footprints, motivated us to ask whether input coverage over genes or promoters correlated with expression level.

**Figure 4:**
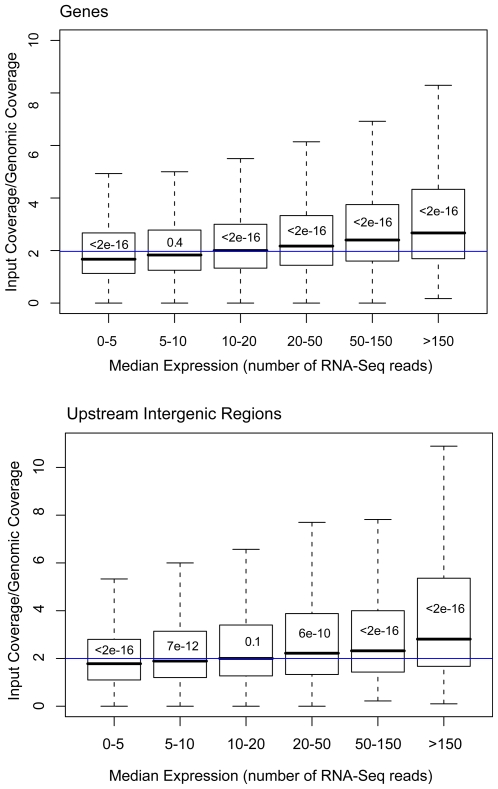
Input-Seq coverage, as a function of gene expression. Boxplots of input-Seq read coverage, normalized to non-crosslinked genomic reads, as a function of the level of gene expression. Input-Seq coverage was binned by expression level, based on RNA-Seq read densities from genome-wide transcription profiling (Nagalakshmi U., 2008). The upper panel shows the boxplots for genes, and the lower panel for intergenic regions. Wilcoxon-Mann-Whitney p-values comparing input coverage distribution at each expression level to genome-wide DNA, are shown within each boxplot. The blue lines indicate the genome-wide median input-Seq read coverage (1.9 for coding and 2.0 for intergenic regions).

We calculated input coverage for genes, pairing it with the median expression level of the gene, based on the published RNA-Seq of *S. cerevisiae* [21]. Compared with genome-wide coding coverage, low-expression genes tended to have significantly fewer reads (Wilcoxon-Mann-Whitney p = 2×10^−16^). The coverage gradually increased, tracking expression level, with much higher read densities for the most highly-expressed genes (Wilcoxon-Mann-Whitney p = 10^−16^) (Figure 4, upper panel). Upstream of the genes themselves, the intergenic regions showed a similar pattern of increased coverage correlating with higher expression of the downstream gene (Figure 4, lower panel).

### Variation in coverage was reproducible in different input-Seq experiments

**Figure 5:**
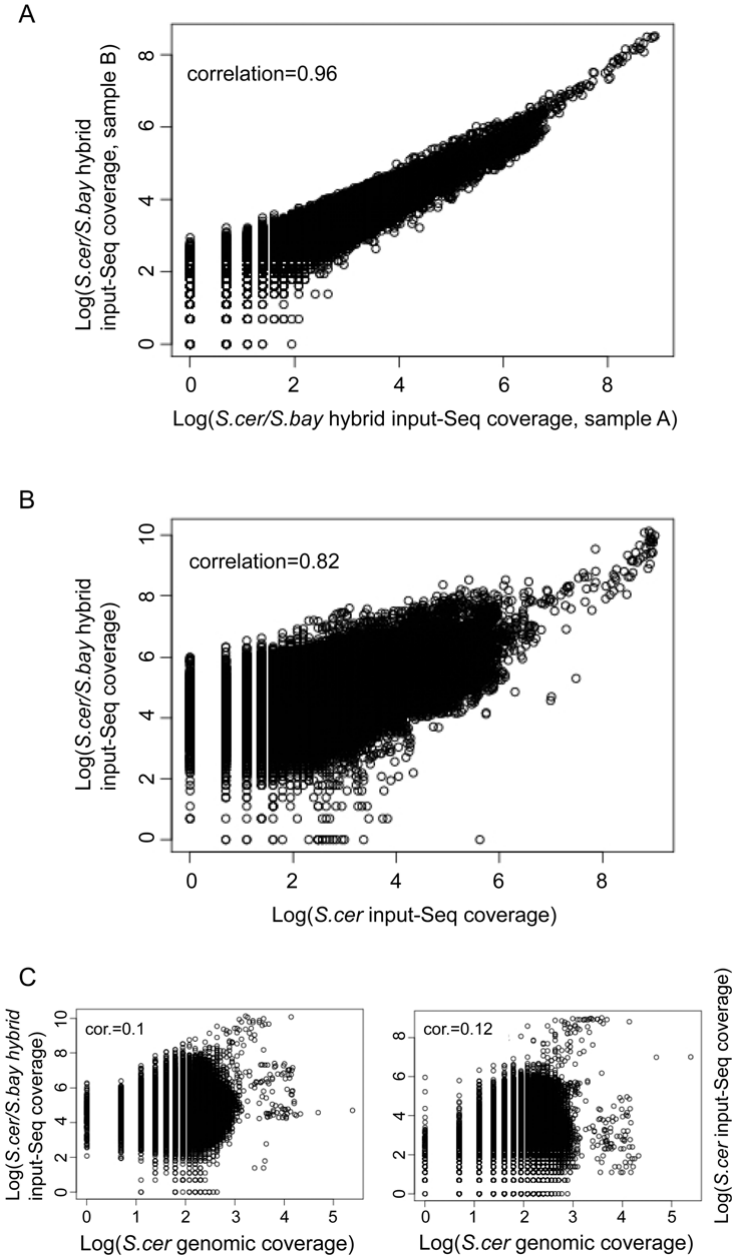
Reproducibility of input-Seq coverage patterns across different strains and experiments. Scatter plots, comparing position-by-position across the genome the sequence read densities between different experiments. A. Comparison of input-Seq read counts between two distinct but nearly-isogenic *S. cerevisiae/S. bayanus* hybrid diploid strains. The samples were prepared in parallel in the same laboratory, as described in the Materials & Methods. B. Comparison of input-Seq read counts between samples from *S. cerevisiae* and *S. cerevisiae/S. bayanus* hybrid, prepared in different laboratories. C. Comparison of genomic sequence read counts to input-Seq read counts (*S. bayanus/S. cerevisiae* hybrid comparison in the left panel, and *S. cerevisiae* input-Seq comparison in the right panel).

To test the reproducibility of the input-Seq coverage patterns, we analyzed input-Seq reads from four additional samples. These four inputs were from nearly isogenic strains of *S. cerevisiae/S. bayanus* hybrid diploids (differing only at two marker loci). These samples were prepared and sequenced in a different laboratory (UC-Berkeley's Vincent Coates Genome Sequencing Lab) from the one that was used for the analyses mentioned above (Yale University Medical Center). The read densities across the genome were tightly correlated among all pairs of the four samples, with Pearson correlation coefficients between 0.94 and 0.99 (Figure 5A, Figure S1). Given the similarity in all four samples, we summed the by-position read counts for reads mapping to the *S. cerevisiae* genome from the four hybrid strains and compared the coverage to the *S. cerevisiae* input-Seq from above (Dataset S1); there was also a high correlation of 0.82 (Figure 5B). This level of consistency was specific to the input-Seq samples, as the correlation with coverage between the input and genomic samples was 0.1 and 0.12 (Figure 5C).

We repeated the above telomeric and subtelomeric analyses with the *S. cerevisiae/S. bayanus* hybrid input-Seq, reaching the same conclusions (Figures S2, S3). We also worried that differences in the size and number of telomeric repeats between S288C and W303 strains of *S. cerevisiae* could play a role in telomeric input-Seq enrichment. To address this, we compared genome-wide and telomeric input-Seq coverage of *S. bayanus*, normalized to genomic sequence reads from the same *S. bayanus* strain. The telomeric input-Seq coverage was high, similarly to the *S. cerevisiae* results (Figure S4). The analyses in this section suggested that chromatin-associated deviations in sequence coverage were robust and reproducible in different labs and strains.

### Normal chromatin shearing in some of the input-Seq hyper- and hypo-covered regions

Because of our prior discovery of the resistance of silenced chromatin to shearing at *HMR* (Özaydın B., submitted), we tested candidate regions, based on the input-Seq analysis, to determine if variation in the sequence coverage was due to shearing differences in the chromatin. We selected probes to three distinct regions: an under-covered subtelomeric region on chromosome V, an over-covered promoter upstream of the *RPL26A* gene, and an under-covered coding region inside the *TRA1* gene (Table 2).

**Table 2:** Candidate regions assayed for shearing and DNA level.

Region	Chromosome	Start	Stop	Median *S. cer* input-Seq reads	Median *S. cer/S. bay* input-Seq reads	Median genomic reads
Entire Genome	**-**	**-**	**-**	16	126	8
*ACT1*	VI	53,843	53,969	23	127	7
*HMR*a1	III	293,833	294,104	7	47	6
Subtelomere	V	564,442	564,621	6	54	10
*TRA1*	VIII	307,328	307,462	7	61	7
*RPL26A* Promoter	XII	818,802	818,971	46	238	7

Chromosome positions of the probes used in the DNA blots to test for shearing differences. The median input-Seq and genomic sequence reads counts are over the indicated intervals for each region. The same five regions were tested in the Q-PCR experiments for their DNA levels in input and genomic samples.

**Figure 6:**
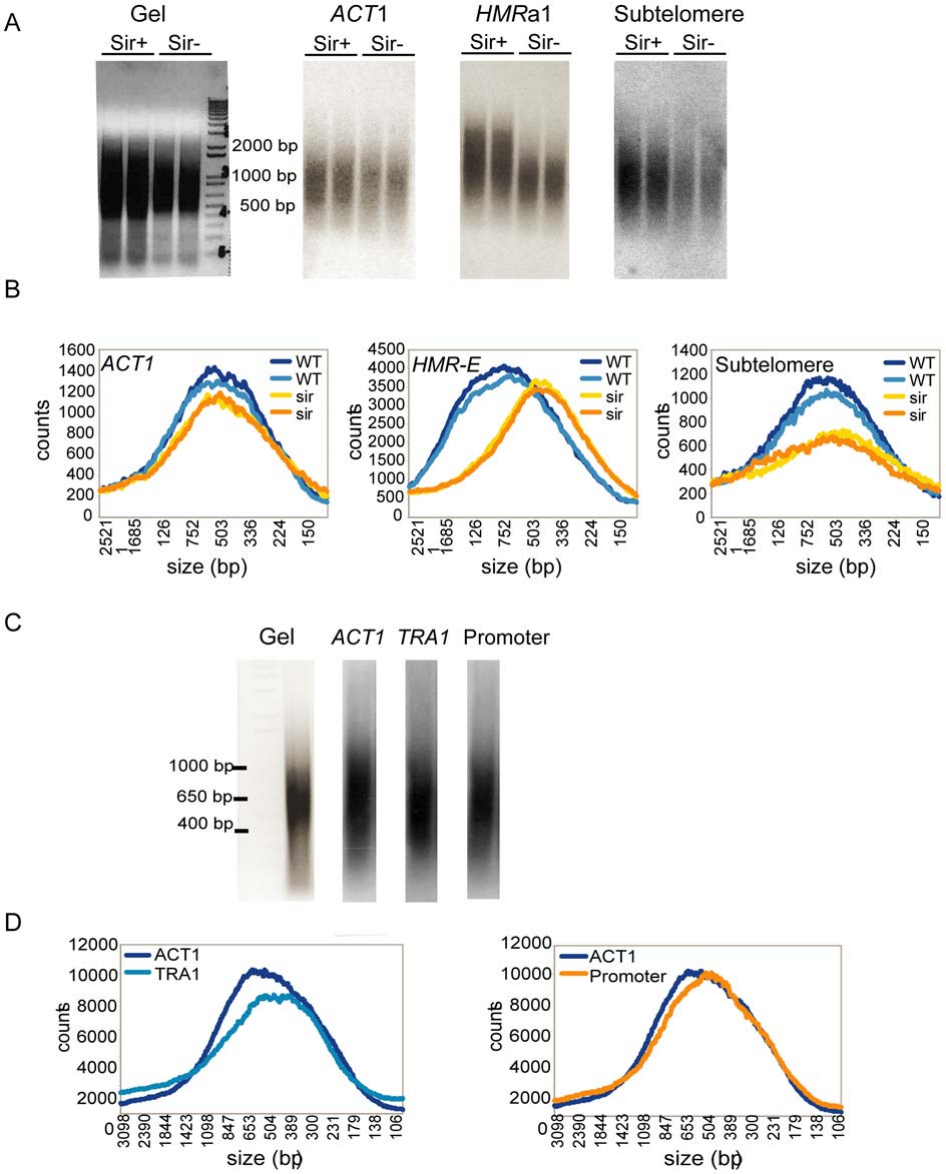
Comparison of shearing in candidate regions. A. Gel image for samples prepared from wild-type and *sir1Δ, sir2Δ* strains and the blots hybridized for *ACT1*, *HMR*a*1*, and the input-Seq under-covered sub-telomeric region. B. The data in the blots were quantified by analyzing the signal intensity for each fragment and plotted as counts (y-axis) against fragment size (x-axis). The plots compare the size distribution of Sir+ and Sir− cells for each probe. C. Gel image for samples prepared from wild-type strains only and the blots hybridized for *ACT1* (average input-Seq coverage), *TRA1* (under-covered), and the over-covered promoter. D. Plots, as in B, comparing size distributions of *ACT1* as a control to the *TRA1* and promoter distributions.

In both Sir+ and Sir− strains, we probed cross-linked and sheared DNA for *HMR*a1, the chromosome V subtelomeric region, and *ACT1* as a control (Figure 6A). As expected, DNA inside the *ACT1* locus sheared similarly in the strains with and without silencing, whereas *HMR*a1 sheared less well in the wild-type strain than in the Sir− strain, as repeated elsewhere (Özaydın B., submitted). Like *HMR*, the under-covered subtelomeric region distribution showed a slight shift toward longer fragments in the wild-type strain, compared to the shorter fragments in the Sir− strain (Figure 6B). In the wild-type strain with intact silencing, the average sheared fragment size for *ACT1* was 752 base pairs, compared with 897 bp for *HMR*a1 and 858 bp for the subtelomeric locus. These data suggested that, similarly to the *HMR* cassette, the Sir−dependent heterochromatization of the subtelomeric regions resulted in shearing resistance, presumably causing under-coverage of these regions in deep sequencing.

Next, we checked the *TRA1* gene (under-covered) and the *RPL26A* promoter (over-covered). These two loci are not bound by Sir proteins and hence were not expected to change in shearing size in Sir− cells. However, it was still possible that they may have different shearing properties for other reasons that would cause variations in their sequence coverage. Therefore, we probed these two candidate regions, along with *ACT1*, to DNA from the wild-type strain (Figure 6C). Both the promoter and *TRA1* showed similar shearing tendencies to *ACT1* (Figure 6D). If shearing resistance were responsible for the reduced coverage of *TRA1* in input-Seq, longer fragments would be expected in our blots, similarly to *HMR* and the chromosome V subtelomeric region. However, the size distribution for *TRA1* slightly shifted toward smaller fragments, relative to *ACT1*, and this was the opposite of what would have been expected based on the input-Seq coverage (Figure 6D, left panel). The shearing of the *RPL26A* promoter was virtually indistinguishable from the shearing of *ACT1* (Figure 6D, right panel). These results suggested that differences in input coverage at these loci were caused by chromatin effects other than those affecting the shearing.

### Variation in input-Seq coverage of the candidate regions was due to chromatin

**Figure 7:**
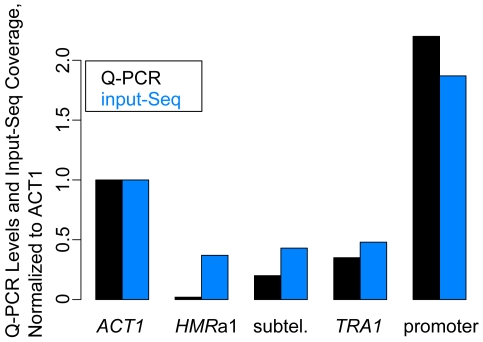
Comparison of DNA levels in candidate regions. Plots of Q-PCR DNA levels (black) in the *S. cerevisiae/S. bayanus* input sample and the corresponding median of sequence reads from the input-Seq dataset (blue). The input Q-PCR measurements are normalized to the genomic Q-PCR results for each region, plotted relative to the *ACT1* input/genomic Q-PCR result. The input-Seq read counts are also normalized to the *ACT1* input-Seq coverage of 127 (genome-wide median coverage for *S. cerevisiae/S. bayanus* input-Seq was 126×).

Given the similar shearing of *ACT1, TRA1* and the *RPL26A* promoter, as described above, we asked whether chromatin structures can influence the concentration of molecules at specific loci in input sample preparation. We used Quantitative PCR (Q-PCR) on input and genomic samples to compare relative amounts of DNA from the regions of interest. For each of the five regions (Table 2), we measured the number of DNA molecules in input and genomic samples, and then normalized the input levels by the genomic results. The Q-PCR results matched closely the input-Seq coverage variation: the three input-Seq under-covered regions (*HMR*a1, subtelomeric region, *TRA1*) had lower Q-PCR levels than *ACT1*, and the input-Seq over-covered *RPL26A* promoter had higher Q-PCR levels than *ACT1* (Figure 7). These quantitative measurements were independent of any technical deep sequencing biases, implying that there were position-specific differences in DNA content, even when shearing was normal.

### GC composition correlated with coverage by genomic and input reads, independently of chromatin structure

**Figure 8:**
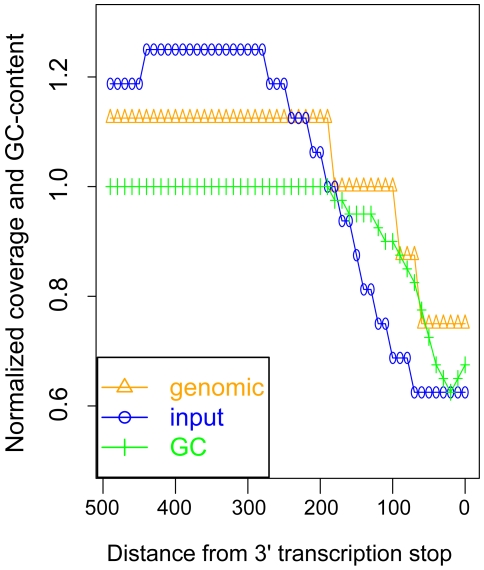
GC-content and sequence read coverage of transcript 3′ ends. Median-normalized sequence read counts from genomic and input samples, and normalized GC-content, as a function of distance from 3′ transcription end sites. The sequence read densities and GC-content were calculated in 10 base-pair intervals, upstream of the 3′ ends.

A sequencing bias toward higher read density in GC-rich regions has been demonstrated in the Illumina-based deep sequencing of the genomes of the plant *Beta vulgaris* and the bacterium *Helicobacter acinonychis* [22]. We observed a similar bias in our samples, which motivated us to normalize all of our input coverage by the genomic sequence reads. Critically, the overrepresentation of sequence reads in GC-rich DNA can be misleading because many genomic features have peculiar GC-compositions, deviating strongly from the genome-wide average of 38% GC-content. For example, centromeres, with 23% average GC-content, had 6× coverage by input reads instead of 16×, and 5× coverage by genomic reads instead of 8×. Also, across all genes, 3′ transcription end sites had an average GC-content of 25%, correlating with a two-fold reduction in coverage for both genomic and input samples (Figure 8).

## Discussion

Heterochromatin is refractory to the activity of several enzymes, including restriction enzymes, DNA methylases and the *HO* endonuclease [23]–[25]. However, it has been previously assumed that physical manipulation of DNA *in vitro* by high energy methods such as sonication is unaffected by biological properties affecting the chromatin under study. This study puts an end to that assumption, with new properties of chromatin revealed in the deep sequencing of samples previously considered as merely controls of other experiments. We discovered that input-Seq coverage differs widely for many distinct positions, including silenced subtelomeric DNA, telomeres, protein-binding sites, and highly transcribed genes and promoters. Such differences will significantly influence interpretation of ChIP experiments, an issue that was previously unrecognized. These differences can also be exploited to detect unusual chromatin states.

By comparing coverage of sequence reads from sheared chromatin samples to those from sheared genomic DNA, we were able to separate technology-related sequencing biases from biologically meaningful effects. The most under-covered regions were heavily biased towards subtelomeric regions which are subject to silencing in yeast, similarly to *HML* and *HMR* [12]–[14]. This analysis supported the hypothesis that silencing interfered with shearing of DNA. In contrast, the DNA inside the telomeres was vastly over-represented in the sequenced input sample. Yeast telomeres, as in other organisms, are specialized structures, with highly repetitive sequences, coated by a variety of proteins [26]. The over-representation of sequence reads on chromosome ends was specific to the sheared chromatin sample and was not observed in the sheared genomic DNA. Hence, peculiar DNA sequence composition inside the telomeres could not explain the over-representation of input sequence reads in these regions.

We observed striking differences in the coverage of protein-bound sites. The sequences around transcription factor binding sites and DNase I footprints had higher coverage than intergenic or coding DNA. The read density over genes and their promoters also correlated with the transcription level of the gene–a high expression level was associated with an increase in read density, and a low expression with a decrease. The increased coverage of the binding sites and DNaseI footprints, and the correlation between high coverage and high RNA levels may have reflected the frenetic activity of nucleosome remodelers, transcription factors, general transcription machinery, and RNA polymerases. It is noteworthy that the only two transcription factors whose binding sites were not enriched in input reads were Ste12 and Dig1. Both are involved in the mating and invasive growth pathways [27], and therefore, would probably have been inactive under the rich media (YPD) conditions in which the cells were grown in preparation for input-Seq.

In testing candidate hyper- and hypo-covered input-Seq regions, we observed changes in shearing similar to *HMR* **a1** only in the under-covered subtelomeric region. Shearing appeared to be normal inside the poorly-covered *TRA1* gene and the over-covered *RPL26A* promoter that we analyzed. These results suggested that chromatin states can also influence input-Seq coverage through effects other than shearing. Indeed, Quantitative PCR (Q-PCR) measurements for the above regions showed similar variation in DNA content of the input sample, as we observed in the input-Seq coverage. It is likely that the chromatin states of the telomeric structures, promoters, and genes, lead to differences in the efficiency of isolation of chromatinized DNA, prior to the shearing step or during the reversal of crosslinking.

Chromatin immunoprecipitation, in conjunction with tiled Q-PCR, is often used to establish the extent of spreading along a chromosome for proteins of interest. If a locus is refractory to shearing and/or is inefficiently isolated due to the chromatin state, a ChIP-based localization of a protein in such a region would exaggerate the apparent interval over which that protein interacts with chromatin. Conversely, a higher susceptibility to shearing or better isolation may result in an under-estimate of the spreading. Particularly for ChIP-Seq studies, our observations of the pervasive inhomogeneity of coverage in the input sample highlighted the need to normalize the sequence read counts from ChIP samples to the input control counts. Many studies currently lack sheared chromatin input sequencing data, and the analyses from these studies are likely to have increased false positives and false negatives.

In addition to the effect of the chromatin structures on ChIP studies, our study re-emphasizes the importance of normalizing deep sequencing results to the sequence reads from genomic DNA. Bias in GC-content and other sequence composition patterns can produce dramatic peaks or troughs in coverage, as we observed over centromeres and across transcripts, potentially leading to mistaken inferences about the underlying biology. These biases would affect ChIP-Seq studies, and would also confound interpretation of RNA-Seq and copy-number variation detection using high-throughput sequencing technologies.

As more ChIP-Seq experiments with appropriate input controls are performed, the deviation in coverage is going to become an increasingly powerful way to identify distinct chromatin states, as long as the raw data from such studies remain available. We were already able to pinpoint specific regions, with decreased or increased read counts defining domains hundreds of base pairs long. Given the highly reproducible results that we observed in the different input-Seq experiments, as more ChIP-Seq input controls for the same species become available, it will become possible to detect chromatin differences at specific loci with increasing resolution. The chromatin-related variation in ChIP experiments is likely to be pervasive across taxa.

## Materials and Methods

### Input-Seq datasets, mapping, and filtering

The *S. cerevisiae* formaldehyde cross-linked sheared input samples were prepared as described [28]. *S. cerevisiae* samples were sequenced using the Illumina Genome Analyzer. *S. cerevisiae/S. bayanus* hybrid diploids were generated by crossing *S. cerevisiae* strain W303 to an *S. bayanus* strain derived from the type strain, CBS 7001 (see [29] for details). The *S. cerevisiae/S. bayanus* input samples were prepared for ChIP analysis by formaldehyde cross-linking and sonication as previously described [30]. ChIP-sequencing libraries were prepared as per the Illumina paired-end library protocol, with modifications as per [28], [31]. Following adapter ligation, 500 bp library inserts were selected on a 2% agarose gel. The genomic library was prepared from the parent *S. bayanus* strain. Libraries were sequenced by 36 bp paired-end reads on the Illumina Genome Analyzer II. The *S. cerevisiae* genomic reads are from the *Saccharomyces* Genome Resequencing Project, and included only the S288C *S. cerevisiae* strain [11], also sequenced using the Illumina Genome Analyzer.

The reads were mapped to the *S. cerevisiae* genome using the MAQ software [32]. Due to poly-A sequencing bias of the Illumina Genome Analyzer, we excluded all reads mapping within 50 base-pairs of a run of 10 or more consecutive adenines or consecutive thymines. The sequences in the rDNA locus (chromosome XII, positions 430,000–520,000) were also not analyzed, as the published SGD genome assembly includes only one of the numerous genomic copies of the rDNA, resulting in artificially inflated coverage of this locus.

All sequence reads from the *S. cerevisiae/S. bayanus* input-Seq and the *S. bayanus* genomic sequencing have been deposited in the NCBI Short Read Archive under accession SRP000997.

### Input/genomic normalization

Every base of the genome was assigned the total number of sequence reads overlapping it, separately for the input and genomic sequence reads. Subsequent normalization and analysis, with the exception of transcription stop site coverage analysis, was performed on median read coverage across 100 bp windows, sliding along each chromosome in 50 bp steps. The median input coverage of each 100 bp interval was divided by the median genomic coverage for the same window. All of the sequence coverage analyses, with the exception of 3′ transcription ends, were done on the 100 bp windows.

### Genome-wide under- and over-coverage analysis

For telomeric sequence designation, we used the annotations for each chromosome from the *Saccharomyces* Genome Database [15]. Subtelomeric regions were defined as sequences within 50 KB of the centromere-proximal telomere edges. The silenced mating loci coordinates (based on flanking genes) on chromosome III are 11,082–15,798 for *HML* and 289,255–297,046 for *HMR*. The percent of 100 bp windows with median input/genomic ratio<0.8 was calculated in 20 KB intervals, at increasing distance from telomeres. Subtelomeric regions excluded telomeric sequences.

### Coverage of protein-DNA interaction sites

Binding site positions were based on [19]. Of the transcription factors whose binding sites were predicted in this study, 37 had forty or more binding sites throughout the *S. cerevisiae* genome. We used the 37 transcription factors to analyze median binding site coverage across all of the binding sites, per factor. The DNase I footprint locations were from http://noble.gs.washington.edu/proj/footprinting/yeast.footprints.bed [20]. Coding and intergenic regions were defined as described below and excluded windows overlapping binding sites or footprints.

### Correlation of gene expression with coverage of genes and intergenic regions

Expression levels were obtained from the genome-wide RNA-sequencing dataset [21]. For each gene, the expression level was defined as the median of all the mapped RNA sequencing reads from that segment. Intergenic regions of *S. cerevisiae* were defined as sequences between transcript ends of all SGD-annotated genes, including uncharacterized, dubious, and coding regions. Transcript ends were defined using the annotations from the RNA-sequencing dataset, to exclude 5′ and 3′ untranslated regions from the intergenic sequence. Intergenic regions between convergently-transcribed genes were excluded. Each intergenic region was paired with the median gene expression of its downstream transcript. For intergenic regions between divergently-transcribed genes, each region was paired with the most expressed of the two genes.

### Comparison of input-Seq experiments

For each 100 bp window with sequence reads from the *S. cerevisiae* input-Seq, median read counts were calculated for each of the four *S. cerevisiae/S. bayanus* hybrid input-Seq experiments. Scatter plots and correlation coefficients were plotted for all pairs of the hybrid input-Seq datasets. Subsequently, for each base of the genome, sequence read counts were summed from all four hybrid input-Seq mapped results. The combined *S. cerevisiae/S. bayanus* read counts were then used to calculate median coverage for the 100 bp windows, and then compared to the *S. cerevisiae* input-Seq coverage in a scatter plot. Comparisons of the genomic coverage to the hybrid input-Seq coverage were based on the summed counts.

### *S. bayanus* telomeric coverage

To identify putative *S. bayanus* telomeric sequence, we used NCBI BLAST [33] without repeat masking (-F F), searching with all of the *S. cerevisiae* annotated telomeric DNA against the Washington University *S. bayanus* assembly [34]. We used e-value cutoff of 0.1 and only accepted matches that were within 5,000 base pairs of a contig end. “Telomeric” coverage was calculated within 500 flanking base pairs of the BLAST matches.

### Genomic DNA Analysis

Whole-cell extracts were prepared as if they were for ChIP analysis as previously described [30]. These extracts were first digested with proteinase K for 2 hours at 37°C and then extracted with phenol-chloroform. After isopropanol precipitation and a 70% ethanol wash, the pellet was resuspended in 50 µl of water. About 10–15 µg of each sample were electrophoretically separated on a 2% agarose gel and then were transferred to Hybond N membrane. Probes of interest were prepared by PCR (Table 3) and then radio-labeled using αP^32^dCTP with Amersham RediPrime Random Prime Labeling System (GE Healthcare). DNA blot analysis was done as previously described [35]. Blots were analyzed with a Typhoon scanner and ImageQuant software. The fragment sizes found at every 0.1 mm of each lane on the gel were calculated using the Invitrogen 1KB+ DNA size ladder.

**Table 3:** Primers used in this study.

Primer set	Sequence
*ACT1*	TGTCCTTGTACTCTTCCGGT
	CCGGCCAAATCGATTCTCAA
*HMR*a1	TGGATGATATTTGTAGTATGGCGGA
	TCCCTTTGGGCTCTTCTCTT
Subtelomere	TGAAACAACGAAGACCTCACCTCG
	AACCGTGAAAGACGGTTTAGCAGC
*TRA1*	TGTTAGATCACCTCACGGCATGGT
	CAGCTTGTGGTGGCAGTAGATGAA
Promoter	TTGCGAAACCGTGCGATGATGTTC
	TGTGTTGGTAGTCATCGAGTCGGA

Primer sequences used to amplify the probes for DNA blots in tests of shearing and also used for the Q-PCR measurements of the genomic and input sample DNA levels at the five loci.

### Q-PCR on candidate regions

For each of the five regions of interest, the same primers were used for the Q-PCR as above (Table 3). The input sample was the *S. cerevisiae/S. bayanus* final 500 bp library–the same one as used for the input-Seq. The genomic sample was also from a W303-based strain (JRY3009). Quantitative PCR (Q-PCR) analysis was done in triplicate for each region, on separate plates for genomic and input samples. Q-PCR was performed on MX3000P machine (Stratagene) using SYBR Q-PCR mix (NEB). The DNA levels were then measured relative to the *ACT1* standard curve. For each primer pair, the DNA level from the input was divided by the DNA level from the genomic sample. The final input/genomic measurements were normalized to the *ACT1* input/genomic result.

### GC bias in coverage of genomic features

GC-content was calculated for each of the 100 bp windows described above, for analysis of centromeres. Across genes, upstream of the 3′ transcription stop sites, input coverage, genomic coverage, and GC-content were calculated in 10 bp windows across all genes. For plots of coverage and GC-content across the genes, median read counts in each 10 bp window were normalized by the genome-wide median reads counts (16 for input-Seq and 8 for genomic), and GC-content was divided by the genome-wide average of 0.38.

### Statistical Analyses

All statistical tests were performed using R [36].

## Supporting Information

Figure S1 **Reproducibility of input-Seq coverage patterns across strains:** Scatter plots, comparing position-by-position across the genome the sequence read densities between different experiments. The six plots show all possible pair-wise comparisons of input-Seq read counts from the four *S. cerevisiae/S. bayanus* hybrid diploid samples.
(3.29 MB TIF)

Figure S2 **Distribution of input-Seq under-covered regions across chromosomes in *S. cerevisiae/S. bayanus* hybrids:** Percent of regions with low input sequence coverage, as a function of distance from telomeres, in 20 KB intervals. The χ^2^ p-values for each 20 KB interval, comparing the fraction of under-covered regions in that interval to the under-covered fraction genome-wide are shown within each plot. The blue line indicates the average percent of under-covered regions, genome-wide (5.9%).
(0.53 MB TIF)

Figure S3 **High input-Seq coverage in telomeres of *S. cerevisiae/S. bayanus* hybrids:** Boxplots of input-Seq read coverage, normalized to non-crosslinked genomic reads, for telomeric and non-telomeric regions. Wilcoxon-Mann-Whitney p-value, comparing input coverage distribution of telomeric to genome-wide DNA, is shown within the telomeric boxplot.
(0.44 MB TIF)

Figure S4 **High input-Seq coverage in telomeres of *S. bayanus*: **Boxplots of *S. bayanus* input-Seq read coverage, normalized to *S. bayanus* non-crosslinked genomic reads, for telomeric and non-telomeric regions. Wilcoxon-Mann-Whitney p-value, comparing input coverage distribution of telomeric to genome-wide DNA, is shown within the telomeric boxplot.
(0.44 MB TIF)

Dataset S1 **Genome-wide input and genomic sequence read coverage:** List with genome-wide positions and median input and genomic sequence read counts for the 100 bp windows.
(2.97 MB GZ)

Table S1 **Input-Seq least-covered regions:** Table of the 300 input-Seq least-covered regions, normalized by genomic read counts.
(0.04 MB XLS)

Table S2 **Input-Seq most-covered regions:** Table of the 300 input-Seq most-covered regions, normalized by genomic sequence reads.
(0.03 MB XLS)
