# Supplementary figures and images for: Impact of Chromatin Structures on DNA Processing for Genomic Analyses
Source: PLoS One. 2009 Aug 20;4(8):e6700. doi: 10.1371/journal.pone.0006700 (PMC2725323; doi:10.1371/journal.pone.0006700)

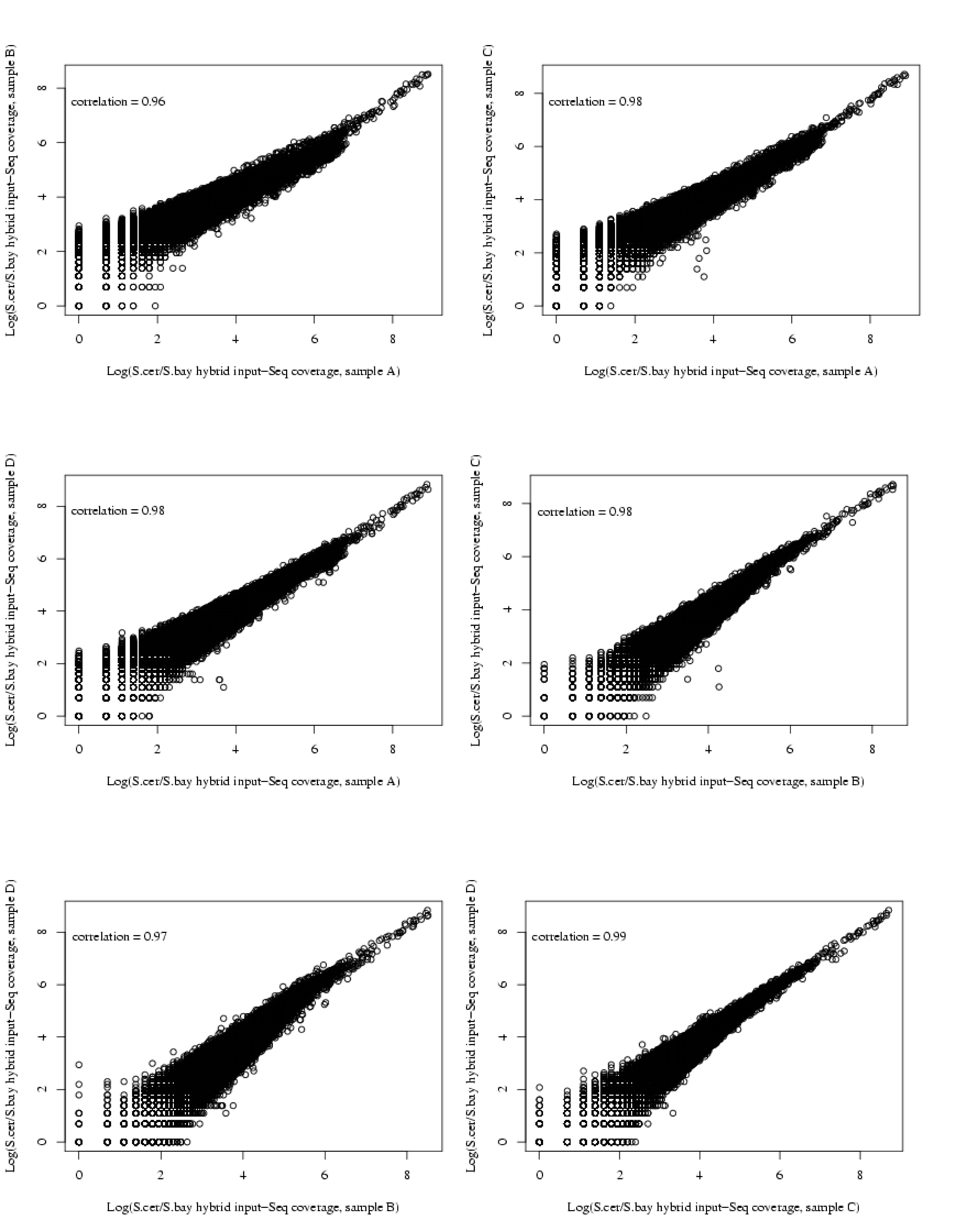

Supplement: Figure S1 — Reproducibility of input-Seq coverage patterns across strains: Scatter plots, comparing position-by-position across the genome the sequence read densities between different experiments. The six plots show all possible pair-wise comparisons of input-Seq read counts from the four S. cerevisiae/S. bayanus hybrid diploid samples. (3.29 MB TIF) [file pone.0006700.s001.tif]

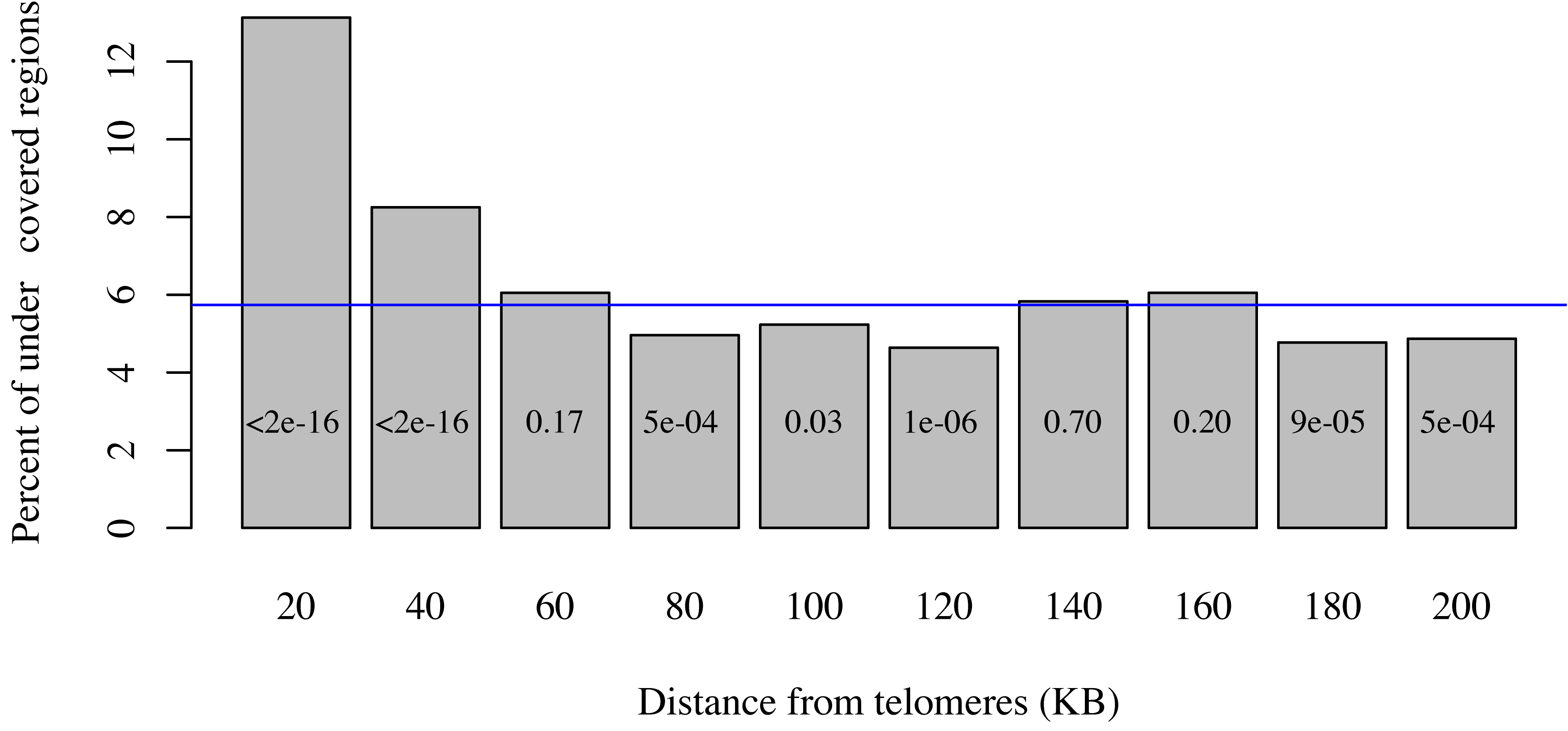

Supplement: Figure S2 — Distribution of input-Seq under-covered regions across chromosomes in S. cerevisiae/S. bayanus hybrids: Percent of regions with low input sequence coverage, as a function of distance from telomeres, in 20 KB intervals. The χ2 p-values for each 20 KB interval, comparing the fraction of under-covered regions in that interval to the under-covered fraction genome-wide are shown within each plot. The blue line indicates the average percent of under-covered regions, genome-wide (5.9%). (0.53 MB TIF) [file pone.0006700.s002.tif]

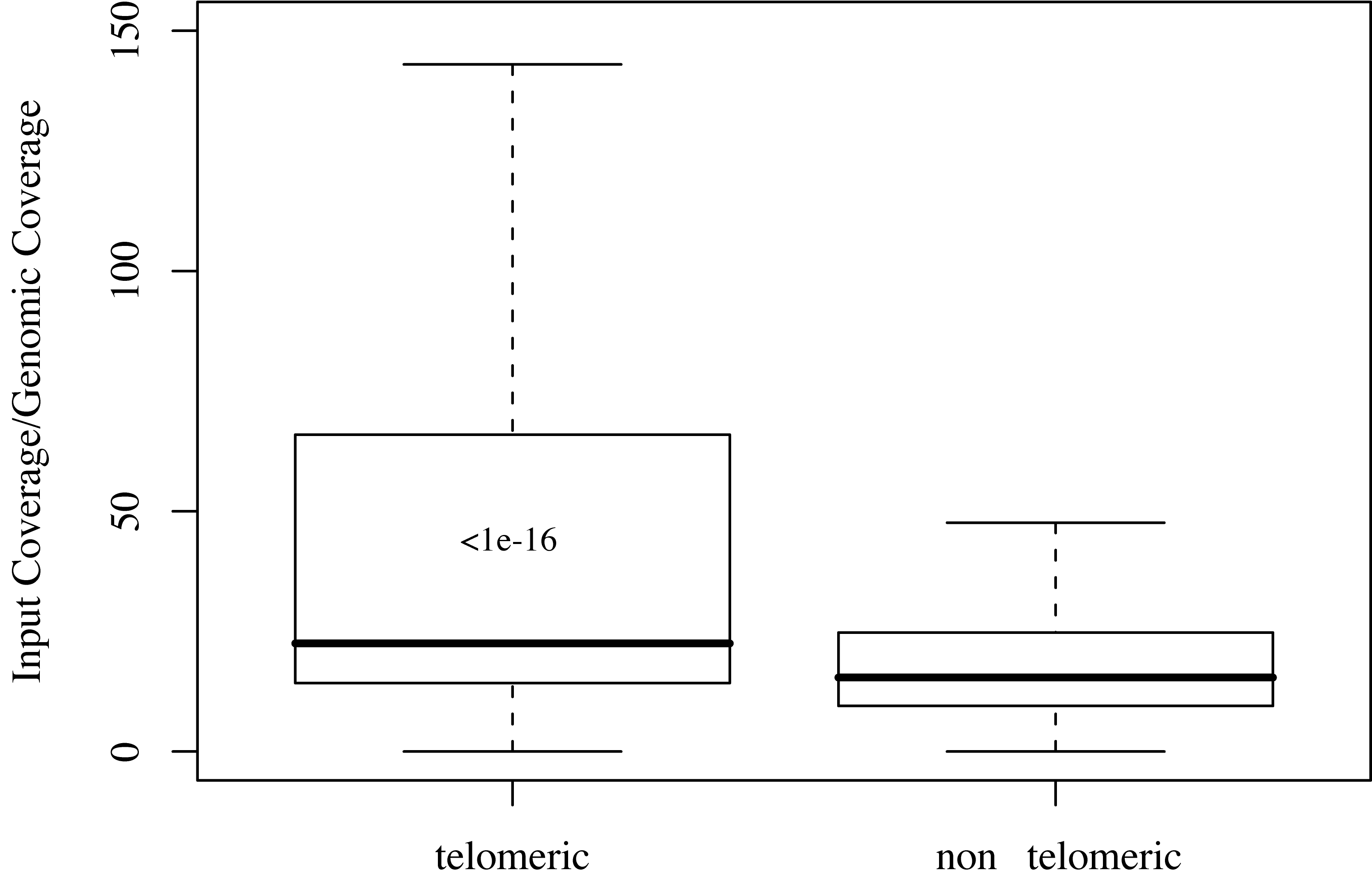

Supplement: Figure S3 — High input-Seq coverage in telomeres of S. cerevisiae/S. bayanus hybrids: Boxplots of input-Seq read coverage, normalized to non-crosslinked genomic reads, for telomeric and non-telomeric regions. Wilcoxon-Mann-Whitney p-value, comparing input coverage distribution of telomeric to genome-wide DNA, is shown within the telomeric boxplot. (0.44 MB TIF) [file pone.0006700.s003.tif]

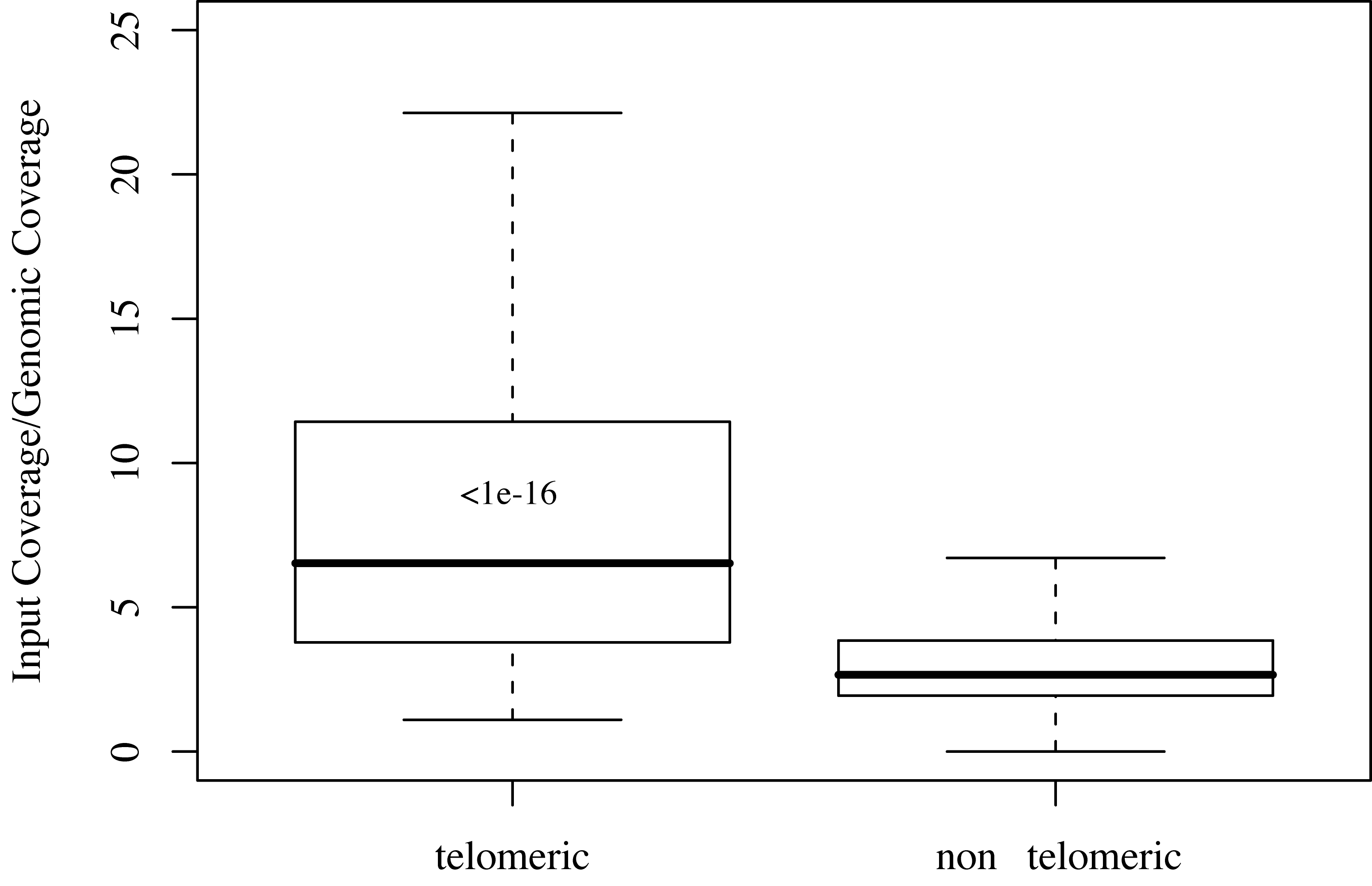

Supplement: Figure S4 — High input-Seq coverage in telomeres of S. bayanus: Boxplots of S. bayanus input-Seq read coverage, normalized to S. bayanus non-crosslinked genomic reads, for telomeric and non-telomeric regions. Wilcoxon-Mann-Whitney p-value, comparing input coverage distribution of telomeric to genome-wide DNA, is shown within the telomeric boxplot. (0.44 MB TIF) [file pone.0006700.s004.tif]
